# Displacement Parameter Inversion for a Novel Electromagnetic Underground Displacement Sensor

**DOI:** 10.3390/s140509074

**Published:** 2014-05-22

**Authors:** Nanying Shentu, Qing Li, Xiong Li, Renyuan Tong, Nankai Shentu, Guoqing Jiang, Guohua Qiu

**Affiliations:** 1 College of Mechatronics Engineering, China Jiliang University, Hangzhou 310018, Zhejiang, China; E-Mails: stnying_2@163.com (N.S.); lixiong@cjlu.edu.cn (X.L.); tongrenyuan@126.com (R.T.); jgqsmile6868@163.com (G.J.); 2 National Engineering Research Center of Advanced Rolling, University of Science & Technology Beijing, Beijing 100083, China; E-Mail: stnk@nercar.ustb.edu.cn; 3 College of Information Engineering, China Jiliang University, Hangzhou 310018, Zhejiang, China; E-Mail: qghfr@163.com

**Keywords:** underground displacement sensor, horizontal displacement, electromagnetic induction, displacement parameter inversion

## Abstract

Underground displacement monitoring is an effective method to explore deep into rock and soil masses for execution of subsurface displacement measurements. It is not only an important means of geological hazards prediction and forecasting, but also a forefront, hot and sophisticated subject in current geological disaster monitoring. In previous research, the authors had designed a novel electromagnetic underground horizontal displacement sensor (called the H-type sensor) by combining basic electromagnetic induction principles with modern sensing techniques and established a mutual voltage measurement theoretical model called the Equation-based Equivalent Loop Approach (EELA). Based on that work, this paper presents an underground displacement inversion approach named “EELA forward modeling-approximate inversion method”. Combining the EELA forward simulation approach with the approximate optimization inversion theory, it can deduce the underground horizontal displacement through parameter inversion of the H-type sensor. Comprehensive and comparative studies have been conducted between the experimentally measured and theoretically inversed values of horizontal displacement under counterpart conditions. The results show when the measured horizontal displacements are in the 0–100 mm range, the horizontal displacement inversion discrepancy is generally tested to be less than 3 mm under varied tilt angles and initial axial distances conditions, which indicates that our proposed parameter inversion method can predict underground horizontal displacement measurements effectively and robustly for the H-type sensor and the technique is applicable for practical geo-engineering applications.

## 1. Introduction

For people throughout the world, geological hazards and disasters are always a potentially catastrophic and costly risk in terms of human lives, property and ecosystem destruction. In the past 100 years, large and super large geological disasters have frequently occurred worldwide [1,2]. Examples include the loess flow triggered by the 1920 Gansu earthquake in China that killed about 0.1 million people, the 1963 Italy Vaiont dam landslide that caused about 3,000 deaths, the collapse and debris flow caused by the 1970 Peru earthquake which led fatalities of more than 25,000, the 1985 Columbian Ruiz volcano mudslides where 22,000 people lost their lives, the m 8.0 Wenchuan earthquake in 2008 China that triggered more than 15,000 geo-hazard occurrences, mainly in the form of landslides, rock avalanches, debris flows and landslide-dammed lakes, and directly caused about 20,000 human deaths, and the debris flow that occurred in Zhouqu County, Gansu in 2010 which resulted in more than 1,700 deaths [1–12].

Underground displacement monitoring can measure the subsurface layered displacement deep inside the studied rock and soil masses. It can effectively determine the deformation mode of geological hazards and geotechnical engineering, locate the position and depth of sliding surfaces, and evaluate the deformation ranges and changing dynamics, so it can provide more comprehensive and reliable information for deformation mechanics analysis, stability/safety assessment, hazard prediction and forecast, prevention and mitigation projects design.

**Figure 1.**
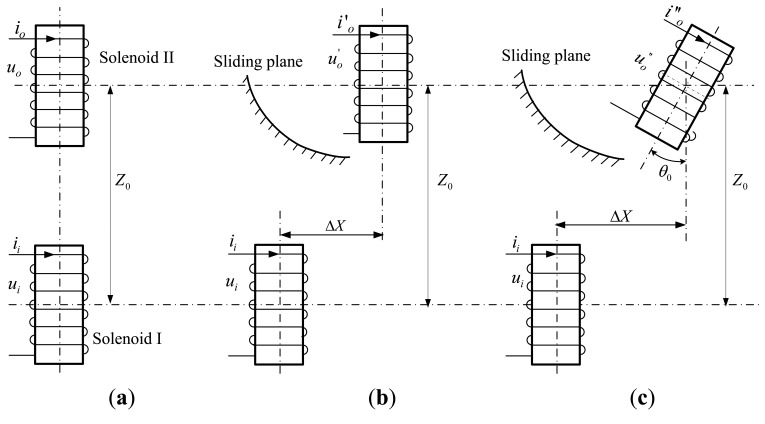
Schematic diagram of the H-type electromagnetic underground displacement sensor. (**a**) Initial geometrical arrangement between Solenoids I and II. (**b**) Occurrence of relative horizontal displacement Δ*X*. (**c**) Occurrences of both relative horizontal displacement Δ*X* and axial tilt angle *θ*_0_.

However, due to the invisibility, complexity and terribleness of underground monitoring objects and conditions, underground displacement monitoring technology has suffered from slow development. Up to now there exist only a few practicable underground displacement monitoring instruments such as inclinometers, settlement gauges, extensometers and TDRs [13–18], with disadvantages such as poor accuracy, high cost, poor durability, low automation capabilities, proneness to electromagnetic interference, vulnerability to signal loss, and non-distributed measurement. In recent years, there has been a lot of exploratory research and testing conducted to develop fiber Bragg grating (FBG)-based sensors to implement effective monitoring of the underground displacements, such as the FBG sensor bar/rod, FBG sensing beam, FBG-based inclinometer, FBG-based extensometer, FBG micro displacement sensor, and so on [19–22]. These FBG-based sensors and instruments have revealed suitable application potential in underground displacement monitoring, due to their specific merits, including light weight, small size, high sensitivity, resistance to corrosion and electromagnetic interference, and easy to realize quasi-distributed measurement. However, some challenges need to be solved before the practical application of FBG sensors in displacement measurement, including the fiber fragility, limited measuring range, low survival rate of embedded FBGs, and the wavelength shifts caused by variations of strain and temperature [23,24]. In former studies [18], we proposed a novel underground horizontal displacement measuring sensor (abbreviated as the H-type underground displacement sensor or H-type sensor) through integration of modern integrated sensing technology with such basic sensing mechanisms as electromagnetic induction and gravity-based inclination measurement. Here we only briefly introduce its main working principle. As depicted in Figure 1, said sensor is mainly composed of two adjacent integrated underground displacement measurement sensing units with the same structure design. For each sensing unit, an air-core solenoid works as the main component that is closely wound by multilayer coils on its hard PVC sleeve. Embedded along the sleeves' inner wall is the integrated sensing circuitry PCB board, which possesses such functions as sine voltage generation (*U_i_*), axial tilt angle measurement (*θ*_0_), mutual inductance voltage measurement (*U_o_*), A/D conversion, and serial communication with the underground displacement measuring central processing unit by RS485 bus.

The geometrical parameters of the proposed H-type sensor are listed in Table 1, and its measuring range for the relative horizontal displacement (Δ*X*) is 0–100 mm.

For the H-type underground displacement sensor, these two sensing units should be vertically pre-buried into the drilling hole and tightly backfilled to make them synchronously deform with the surrounding rock and soil masses before working. During the working process, the lower and the upper sensing units function as the signal excitation unit and signal receiving unit, respectively, and are called Solenoid I and II accordingly. To adapt to the sensor's sensing properties [18], as shown in Figure 1, it is assumed that along with the sliding of surrounding rock and soil masses, a relative horizontal displacement Δ*X* (Figure 1b) or both relative horizontal displacement Δ*X* and axial tilt angle *θ*_0_ (Figure 1c) may occur between Solenoid I and II, but no obvious vertical displacement Δ*Z* happens between them. That is to say, when the relative geometrical arrangement between Solenoid I and II is changed from that shown in Figure 1a to those in Figure 1b,c, the initial axial distance *Z*_0_ between them remains unchanged, so the H-type underground displacement sensor mainly serves for projects as translational sliding, side slope, foundation pit engineering, highway and railway roadbed measurements which mainly require underground displacement monitoring in the horizontal direction.

According to the electromagnetic induction law, if a sine voltage *U_i_* with fixed amplitude and frequency is pre-applied on Solenoid I, the mutual inductance voltage *U_o_* generated on Solenoid II will vary synchronously with the occurrences of relative horizontal displacement (Δ*X* ≠ 0, *θ*_0_ = 0) as shown in Figure 1b or a combination of horizontal displacement and tilt angle (Δ*X* ≠ 0, *θ*_0_ = 0) as shown in Figure 1c. Meanwhile, the sensor's tilt measuring integrated circuit can figure out the relative axial tilt angle (*θ*_0_) between Solenoid I and II in real-time, so the sensor can reversely derive the relative horizontal displacement Δ*X* between these two solenoids according to the output variations of mutual inductance voltage (*U_o_*) and tilt angle (*θ*_0_) under the given geometrical parameters for these two solenoids (e.g., diameter *d*, length *a*, winding coil turns *w*) and the initial relative position between them. Notably, in accordance with the assumption that the H-type sensor is able to deform synchronously with the soil/rock mass around, we can take the relative horizontal displacement Δ*X* between Solenoid I and II on behalf of the H-type sensor's measuring underground horizontal displacement at some given depth (or the sensor's buried depth) within the monitored rock or soil mass.

**Figure 2.**
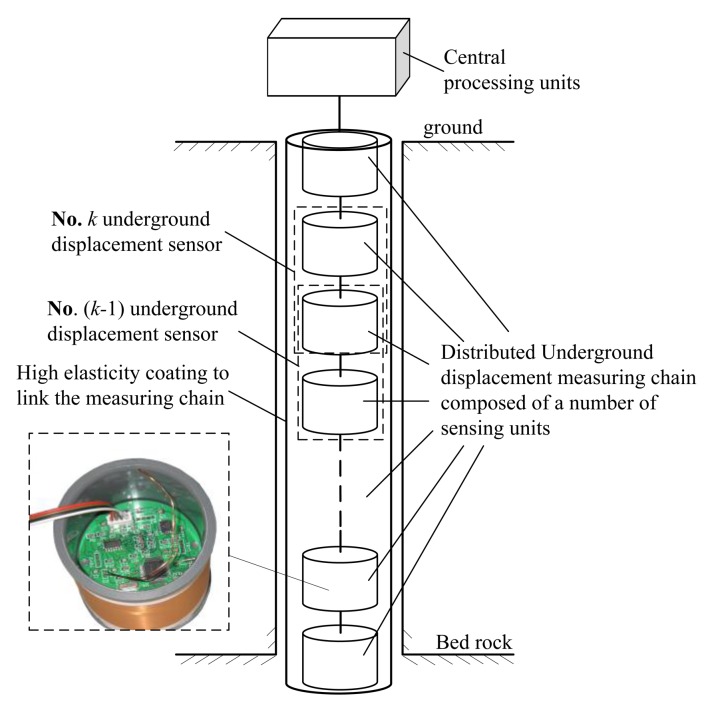
Schematic diagram of the distributed underground displacement measuring apparatus made up of a series of electromagnetic underground displacement sensors.

Centered by the H-type underground displacement sensor and combined with the design idea of a linear sensor array, we have further proposed a novel distributed underground displacement measuring apparatus. As Figure 2 shows, it is composed of a group of H-type sensors in a linear array distribution and an underground displacement measuring central processing unit [18]. Each H-type sensor is employed to measure the relative horizontal displacement and tilt angle at some given depth within the studied rock and soil masses. Meanwhile, the underground displacement measuring apparatus, functioned as an underground displacement measurement chain, can measure the cumulative underground displacement and sliding angle from the surface till to the bedrock within the monitored mass. Compared to the existing underground displacement monitoring sensors/instruments, our proposed electromagnetic underground displacement measuring sensor and apparatus features a simple structure design, low monitoring cost, high measurement accuracy, big measurement range, and good long-distance data transmission capability, to achieve an automatic measurement of the subsurface displacement and sliding direction within the monitored mass.

Meanwhile, after comprehensive research on various factors and parameters affecting the sensing characteristics of the H-type sensor, a mutual inductance voltage measurement model called the Equation-based Equivalent Loop Approach (EELA) was put forward [18] through technical fusion of the equivalent loop modeling of solenoids, the electromagnetic field formula derivation and mathematical multiple integral approximation. The model can quite accurately evaluate the complicated relationship among the H-type sensor's mutual inductance voltage output, the measuring parameters including the underground horizontal displacement and tilt angle between two adjacent sensor units (*i.e*., Solenoid I and II), and the shape and geometrical parameters of sensor units (e.g., the diameter, length and coil turns of Solenoid I and II, and the initial axial distance between them). Moreover, through comparative studies of changing characteristics of mutual inductance voltage between the sensor experimentally measured and EELA simulated data in MATLAB, EELA has been proven to be an competent model for characterizing the H-type sensor's detection characteristics towards the underground horizontal displacement and tilt direction with quite high estimation accuracy and calculation efficiency and good suitability for hardware implementation.

In our previous work [18], the EELA model was described in detail. Here we give only a brief review of its working principle. Generally speaking, EELA is essentially a kind of semi-analytic approximate calculation with the following basic modeling steps:

Step 1: as our previous studies have proven, firstly, it's more efficient to study the horizontal displacement parameter (Δ*X*) in terms of the mutual inductance *M* between Solenoid I and II rather than the mutual inductance voltage *U_o_* generated on Solenoid II. Secondly, *M* is strictly proportional to *U_o_* with the following relationship being satisfied:
  (1) $$U_{o}=\frac{1}{R}\frac{dU_{i}}{dt}M$$
  where *R* is the equivalent resistance of Solenoid I, and *U_i_* is the sine input voltage applied on Solenoid I.
Step 2: after neglecting the helicity and ununiformity of the circular coils wound on Solenoids I and II, each solenoid may be replaced by two “equivalent current loops”, whose diameter and position are determined according to the following rules: the magnetic field generated by these two loops is nearly equal to that generated by the original Solenoid I and II under the counterpart magnetic potential. That is, the mutual inductance between Solenoids can be equivalent as:
  (2) $$M=w^{2}(M_{13}+M_{14}+M_{23}+M_{24})/4$$
  where, *M*_13_, *M*_14_, *M*_23_, and *M*_24_ are the mutual inductances between two equivalent loops 1 and 3, 1 and 4, 2 and 3, 2 and 4, respectively.
Step 3: full applying related electromagnetic field theory and equations to deduce the analytic expressions of mutual inductance *M*_13_, *M*_14_, *M*_23_ and *M*_24_.

For example, when Solenoid I and II are arranged in the parallel-axial state as depicted in Figure 1b, we can deduce the following mutual inductance analytic solution to *M_ij_*:

(3) $$M_{ij}=\frac{\mu_{0}\pi R_{i}R_{j}}{4}\sum_{n=1}^{\infty }{\left(-1\right)}^{n+1}\frac{n}{n+1}{\left[\frac{(2n-1)!!}{(2n)!!}\right]}^{2}\lambda_{ij}^{2n+1}P_{2n}(\eta_{ij})$$

where, *i* = {1,2}, *j* = {3,4}, $\;r_{ij}=\sqrt{X_{ij}^{2}+Z_{ij}^{2}}$, λ = 2*R_i_*/*r_ij_*, η*_ij_* = *Z_ij_*/*r_ij_*, *X_ij_* and *Z_ij_* are the horizontal and vertical distance between equivalent Loop *i* and Loop *j*, *R_i_* and *R_j_* are the radius of Loop *i* and *j* respectively, *P*_2_*_n_*(η*_ij_*) is the 2*n*-order Legendre polynomials of argument η*_ij_*, μ_0_ = 4π × 10^−7^*H*/*m* is the free space permeability.

It deserves emphasis, however, that the monitoring objects of H-type sensors are invisible and complex subsurface rock and soil masses with highly nonlinear characteristics, and the sensor's output is not directly a measured underground displacement and tilt angle but a mutual inductance voltage that lacks a clear physical meaning. Moreover, the specialized EELA mutual inductance voltage measuring model is quite abstract and complex. Hence, another key research topic in underground displacement measurement is how to use our proposed EELA model as the theoretical basis of underground horizontal displacement measurement to establish an efficient and practical underground displacement parameter inversion algorithm, which can directly convert the real-time output of mutual inductance voltage into the H-type sensor's measuring parameters-the relative horizontal displacement and tilt angle at different subsurface depth within the monitored mass.

For these, this paper proposes an underground displacement inversion method based on the EELA forward modeling and approximate optimization inversion theory. It utilizes the above introduced semi-analysis EELA model as the forward model to generate the reference signals of the mutual inductance voltage, which is then used as entry data of the inversion system, together with the measured data of mutual inductance voltage and axial tilt angle of the H-type sensor. Through further application of a comprehensive optimization algorithm, the inversion system can finally realize the underground horizontal displacement parameter inversion for the H-type sensor with fairly high prediction precision.

## 2. Basic Theory of Parameter Inversion

The parameter inversion method, also called the reverse analysis method in system identification theory, is characterized by the establishment of a reasonable mathematical model to simulate the unknown practical system, and by implemention of an iterative process to modify the model parameter gradually so as to minimize or optimize the error between the model output and the actual output of system in a certain sense [25,26], so parameter inversion is an effective way to deduce one or more initial parameters (such as displacement, speed, geometric parameters and the constitutive model parameters) through establishing and executing the effective inversion models (*i.e*., the system mathematical models or mathematical descriptions), into put which are some field measurable physical parameters (such as stress, strain, load *etc.*) that can reflect the system behaviors or features [27]. An effective parameter inversion process mainly includes the following three elements:

(1) Output data of the unknown system must be collected as accurately as possible;
(2) Mathematical model to simulate the unknown system must be reasonable and effective;
(3) Parameter adjustment algorithm must be as quick and efficient as possible.

According to the differences between calculation methods, the parameter inversion method can be divided into two categories: the analytical method and the numerical method. The analytical inversion method is mainly used for the parameter inversion with simple geometry and boundary conditions and is generally not suitable for parameter inversion under the complex geotechnical engineering conditions. By comparison, the numerical inversion method has better universality. According to the different back analysis process, it can be classified into three types: the backward solution method, the direct/forward inversion method, and the mapping method. Among them, the backward solution method is established on the basis of inverse matrix calculations, so this method is not universal and only suitable for linear inversion problems. The basic idea of the direct/forward inversion method is converting the parameter inverse process into a certain objective function optimization problems. That is, it first establishes an object function, then directly takes on the forward analysis process and executes the iteration process of least error function to modify and optimize step by step the trial value of the unknown function, thus the direct inversion method is mainly featured by a quite large calculation burden, a wide scope of back-analysis adaptation, and the capability of executing parameter inversions for all kinds of linear and nonlinear problems. At present, the parameter inversion method has mainly developed towards two directions: one is some intelligent parameter inversion methods put forward for the purpose of improving the inversion theoretical study depth, such as the genetic algorithm, artificial neural network algorithm, ant colony algorithm, and simulated annealing algorithm [27–30]; the other one is a series of simple but practical inversion approaches mainly aimed at solving some practical engineering problems with the acceptable inverse precision required by the practical engineering projects.

Starting from the practical problems of underground displacement monitoring, this paper utilizes the above mentioned EELA theoretical model as the mathematical model of parameter inversion, and combines it with the optimization inversion method to solve the inverse problems of underground displacement for the H-type underground displacement sensor.

## 3. Underground Displacement Parameters Inversion Method

Firstly, we introduce briefly the optimization inversion method. Generally speaking, it is a direct parameter back analysis approach based on the optimal control principle, whose basic ideas are as follows [31]:

If we denote a point in *s*-dimensional space as *h* = (*p*_1_, *p*_2_, …, *p_s_*), then the whole collection of points satisfying *m_i_* < *p_i_* < *n_i_* comprises a rectangle domain in the *s*-dimensional space and can be denoted as *p*. If the magnitude of target function *J*(*p̄*) is set as a standard to assess whether {*p*} satisfies the actual situation, then the parameter inversion problem is transformed into an optimization search problem, that is, to solve {*p*} when satisfies:

(4) $$J(\bar{p})=\underset{\bar{p}\in P}{\min}E(p),\;\bar{p}\in P$$

Methods to solve this kind of problems are called optimal control methods. Basically, they implement various iterative methods for concrete solving: first to set a group of initial estimates for the parameters to invert, then use the iteratively obtained values to modify the initial value gradually, so as to continuously decrease the value of the objective function *J* (such as the minimum error function) while satisfying certain constraints. The optimized value is further tested with the convergence criteria specified in advance. If it is judged to satisfy the convergence condition, the optimization process ends and the final iteration result is output as parameter inverse value.

One key point in the parameter inversion process is to establish a reasonable and accurate mathematical model to simulate the unknown system. For the H-type underground displacement sensor, thanks to our previous theoretical researches, a mutual inductance voltage measuring theoretical model with relatively high calculation accuracy and suitable for hardware implementation has been established, namely the EELA model, to describe the complex relationship among the H-type sensor's measuring underground horizontal displacement and sliding angle, the sensor's output mutual inductance voltage, and the geometry and shape parameters of sensor units. Therefore, to meet the practical demands of underground displacement monitoring, this paper uses the EELA model as the parameter inversion mathematical model and calls it the forward simulation model. Then, we input the initial model parameters and trial estimate values of inversion parameters into the EELA forward model, and run this model's calculation program to obtain the output sequence of simulated mutual inductance voltage (*i.e*., the theoretical values of mutual inductance voltage), and this process is called as the forward simulation process. On such a basis, the combinations of EELA forward simulation model and optimization inversion algorithm comprise the complete parameter inversion method of underground horizontal displacement for the H-type sensor in this paper. This inversion method is then referred to as the EELA forward simulation-optimization inversion method for convenience of study.

**Figure 3.**
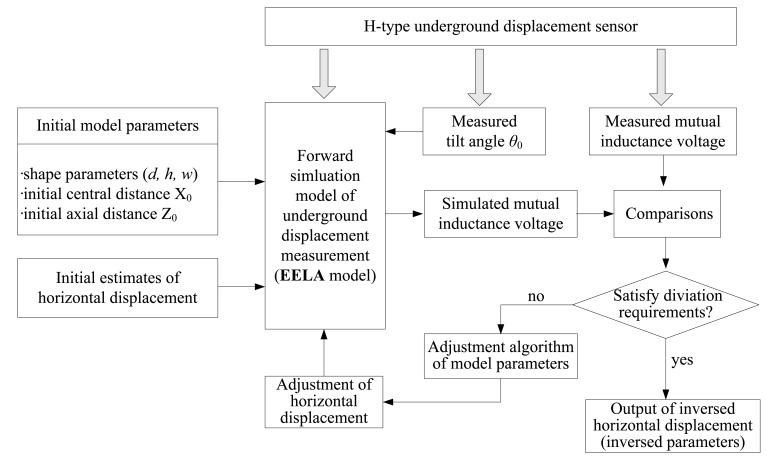
Schematic diagram of displacement parameter inversion for the H-type sensor.

Figure 3 shows a schematic diagram of applying the EELA forward simulation-optimization inversion method to invert the horizontal displacement parameters for the H-type underground displacement sensor. The inversion process can be carried out by the following three basic steps:

(1) Acquisition of mutual inductance voltage data from the H-type sensor. The data may come from two measurable ways: one is the *in-situ* measurement results of mutual inductance voltage when the H-type sensor is buried into the monitored rock and soil masses; another one is the testing values under experimental conditions where the relative tilt angle and the horizontal displacement values between two sensor units are artificially changed so as to record the corresponding variations of mutual inductance voltage. In this paper the latter way is used.
(2) Execution of the EELA forward simulation process, that is, input of the initial model parameters and horizontal displacement estimate values into the execution program of the EELA forward simulation model to generate the corresponding mutual inductance voltage simulated/theoretical values. The initial model parameters include the shape parameters (e.g., length *a*, diameter *d*, and coil number *w* of Solenoid I and II) and initial geometry parameters (e.g., initial axial distance *Z*_0_ and tilt angle *θ*_0_).
(3) Execution of the module parameter adjustment process. That is, compare the simulated values of mutual inductance voltage based on the EELA forward simulation in Step 2 with the measured values of mutual inductance voltage in Step 1 so as to gradually adjust the relative horizontal displacements that are input into the EELA model through the module parameter adjustment algorithm, until the simulated and the measured mutual inductance voltage can meet the required accuracy or reach the minimum discrepancy between them. The final iterative result is then output as the horizontal displacement inversion result.

## 4. Experiments and Evaluations of Underground Displacement Parameters Inversion

To examine and evaluate the above proposed underground displacement parameter inversion method, we will conduct a series of experiments of underground displacement parameters inversion for the H-type underground displacement sensor in this section.

### 4.1. Experiment Setup and Procedure

The H-type sensor's output includes the mutual inductance voltage *U_o_* and relative axial tilt angle *θ*_0_ between two sensor units (Solenoid I and II), and the parameter to invert is the relative horizontal displacement value between them, namely the sensor's measuring underground horizontal displacement at some given underground depth. The related parameter inversion experiment setup and assessment scheme will first be briefly introduced.

#### 4.1.1. Experimental Equipment

**Figure 4.**
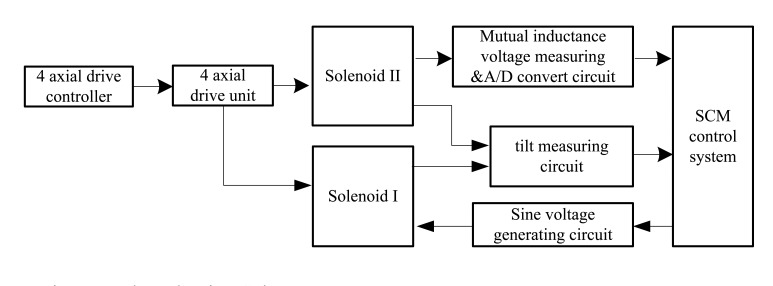
Schematic diagram of the experimental setup.

Experiments were performed on the electromagnetic underground displacement measurement testing platform, which is established at China Jiliang University's Geological Disaster Monitoring Equipment Research Center and has been detailed in our previous paper [18]. Here we only give an overview of it. As illustrated in Figure 4, the testing platform is mainly composed of three parts: (1) the prototype of H-type electromagnetic underground displacement sensor, which is mainly made of Solenoid I, Solenoid II, and their embedded sensing integrated circuits PCB that integrates such functions as sine voltage generation, mutual inductance measurement, ADC, tilt measuring, RS485 communication, *etc.*; (2) 4-axis joint drive device and controller of underground displacement measurement based on PC and step motors. It can drive and adjust the relative horizontal displacement, vertical displacement and tilt angle between Solenoid I and II with the adjustment precision more than 0.1 mm displacement and 0.2° axial angle, respectively; (3) SCM control system. It can implement an automatic measurement and real-time recording of such measuring parameters as the relative horizontal Δ*X*, vertical displacement Δ*Z* and axial tilt angle *θ*_0_ between Solenoid I and II, and the sensor's output of mutual inductance voltage *U_o_*.

#### 4.1.2. Experiment and Evaluation Scheme

The horizontal displacement inversion experiments and evaluation are set up as follows:

(a) Before the experiment, the initial axial distance *Z*_0_ and axial tilt angle *θ*_0_ for the H-type sensor are set as fixed values, and the initial central distance *X*_0_ is fixed to 0. During the experiment, we change point-by-point the sensor's relative horizontal displacement *ΔX_i_* (*i* = 1, 2, …, *m*), and record the corresponding output values of mutual inductance voltage *U_oi_* so as to form the variation sequences of measured mutual inductance voltage *U_o_* = {*U_o_*_1_, *U_o_*_2_, …, *U_om_*} required by the parameter inversion process. Meanwhile, this set of measured horizontal displacement variable series *ΔX*_1_ = {*ΔX*_1_, *ΔX*_2_, …, *ΔX_m_*} can be regarded as the actual (measured) horizontal displacement values to invert.
(b) As the initial model parameters of the H-type sensor, the shape parameters (length *a*, diameter *d*, coil turns *w*, *etc.*) and initial geometric position parameters (initial axial distance *Z*_0_, central distance *X*_0_ and tilt angle *θ*_0_, are all required to be set as the same values of step (a)) are input into EELA forward simulation model. At the same time, a trial estimated sequence of horizontal displacement *ΔX′* = {*ΔX′*_1_, *ΔX′*_2_, …, *ΔX′_m_*} within 0–100 mm variation range and 0.5 mm variation step is input into the EELA model. After that, execution of the EELA forward simulation calculation program can output a set of mutual inductance voltage simulated signal accordingly.
(c) Implementation of the proposed EELA forward simulation-optimization inversion algorithm on every point (*U_oi_*) of the measured induced voltage sequence so as to deduce the corresponding horizontal displacement inversion value Δ*X′_i_*, which, after one-by-one ranked in time, forms the complete horizontal displacement inversed sequence *ΔX′* = {*ΔX′*_1_, *ΔX′*_2_, …, *ΔX′_m_*}.
(d) Comparison of the degree of fitting between the measured horizontal displacement series in step (a) and the inversed horizontal displacement series in step (c), so as to verify the adaptability of the above proposed parameters inversion method for the H-type sensor.

### 4.2. Experimental Results and Evaluations of the Parameter Inversion Method

**Figure 5.**
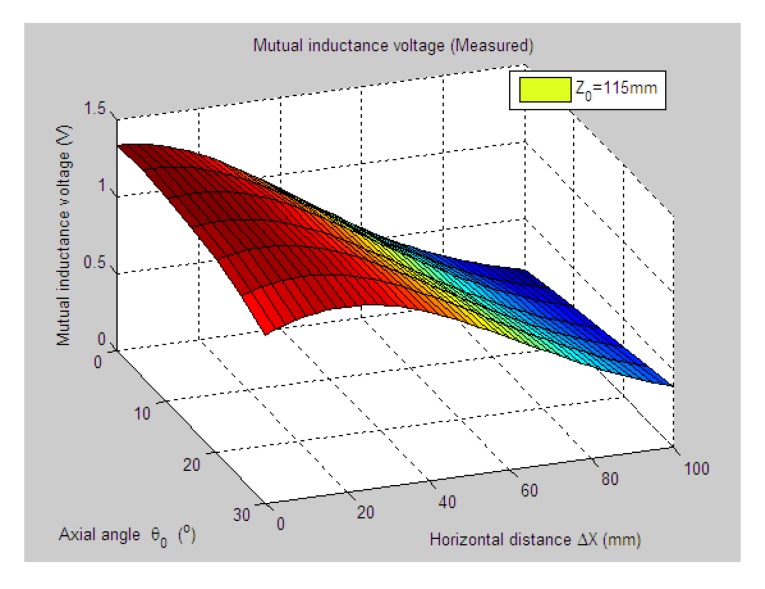
Measured mutual inductance voltage of H-type sensor (*Z*_0_ = 115 mm).

**Figure 6.**
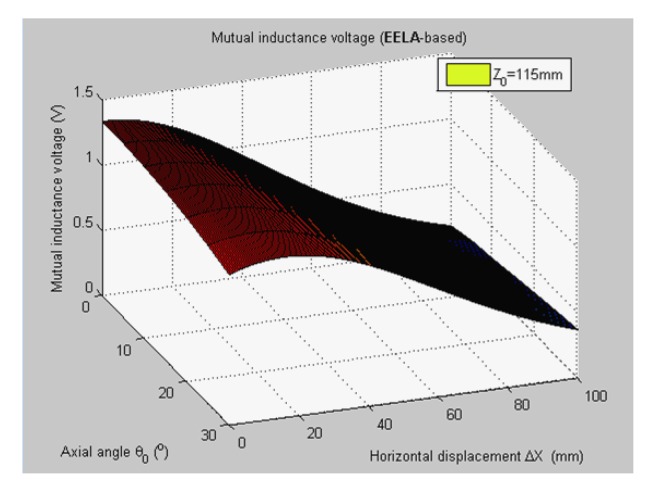
Forward simulated mutual inductance voltage of H-type sensor (*Z*_0_ = 115 mm).

Figure 5 shows 3-D graph of the experimentally measured mutual inductance voltages changed with the simultaneous variations of relative horizontal displacement Δ*X* (0 mm–100 mm) and tilt angle *θ*_0_ (0°, 5°, …, 30°) when specifying the axial distance *Z*_0_ at 115 mm. Figure 6 plots the simulated results of mutual inductance voltage when input the same values of *Z*_0_ and *θ*_0_ into the calculation program of EELA forward simulation model together with the sensor's geometrical parameters (diameter *d* = 70 mm, length *a* = 75 mm, and coil turns *w* = 400), the estimated range and variation step of Δ*X* (0–100 mm and 0.5 mm, respectively).

**Figure 7.**
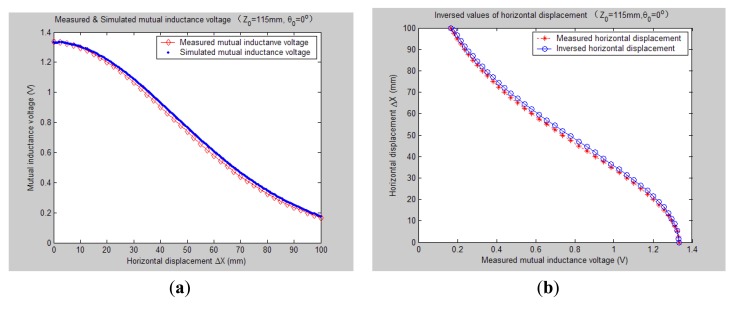
Horizontal displacement inverse results of H-type sensor (*θ*_0_ = 0°, *Z*_0_ = 115 mm).

Figures 7^8^, 9, 10, 11, 12 and 13 display the inversion results of the horizontal displacement Δ*X* after applying the proposed EELA forward simulation-optimization inversion method on the above measured and simulated mutual inductance voltages when fixing the tilt angle *θ*_0_ to 0°, 5°, …, and 30°, respectively.

Table 2 gives both the relative and absolute inversion deviation averages between the inversed and measured horizontal displacement under the above tilt angles.

After a detailed analysis on the above table and figures, it can be concluded that: during the process of varying *θ*_0_ from 0° to 30°, there only exist small overall deviations between the modeling inversed and experimentally measured horizontal displacement, and good curve fitness has displayed between them. It can be seen that when *θ*_0_ is varied from 0° to 30°, the relative and absolute mean inversion deviations of horizontal displacement are ranged between −1.54–1.06 mm and 0.32–2.50 mm, respectively. These experimental results initially show that it is reliable and effective to applying the proposed “forward simulation-optimization inversion method” on the underground horizontal displacement inversion for the H-type sensor with quite acceptable prediction accuracy and stability.

**Figure 8.**
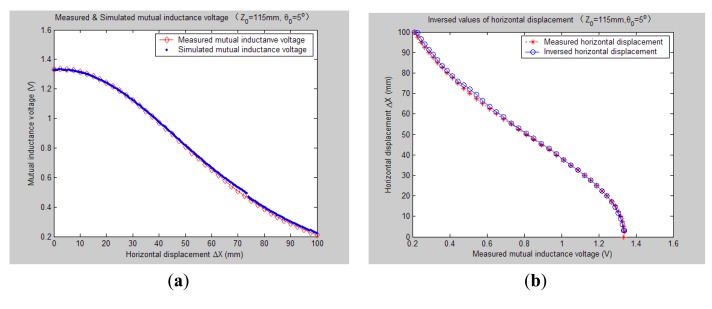
Inversed horizontal displacement of H-type sensor (*θ*_0_ = 5°, *Z*_0_ = 115 mm).

**Figure 9.**
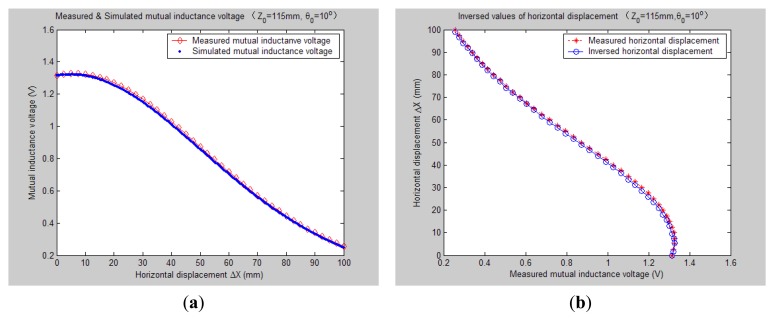
Horizontal displacement inverse results of H-type sensor (*θ*_0_ = 10°, *Z*_0_ = 115 mm).

**Figure 10.**
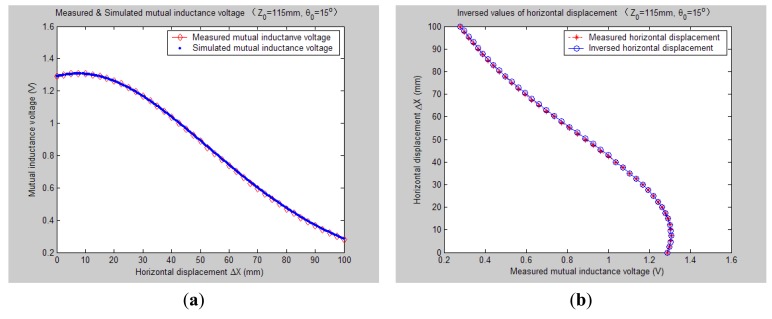
Horizontal displacement inverse results of H-type sensor (*θ*_0_ = 15°, *Z*_0_ = 115 mm).

**Figure 11.**
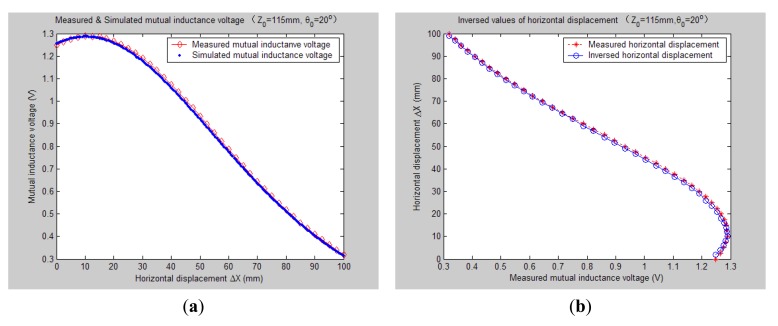
Horizontal displacement inverse results of H-type sensor (*θ*_0_ = 20°, *Z*_0_ = 115 mm).

**Figure 12.**
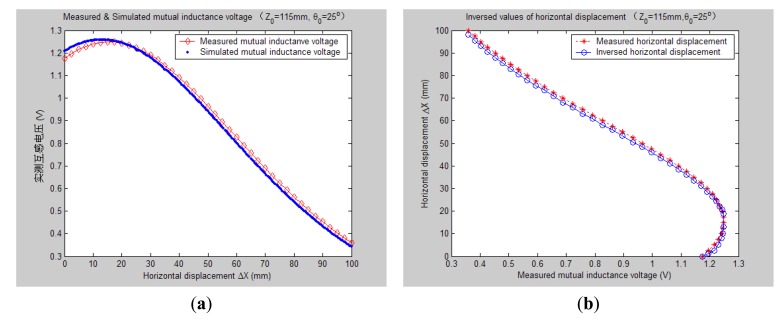
Horizontal displacement inverse results of H-type sensor (*θ*_0_ = 25°, *Z*_0_ = 115 mm).

**Figure 13.**
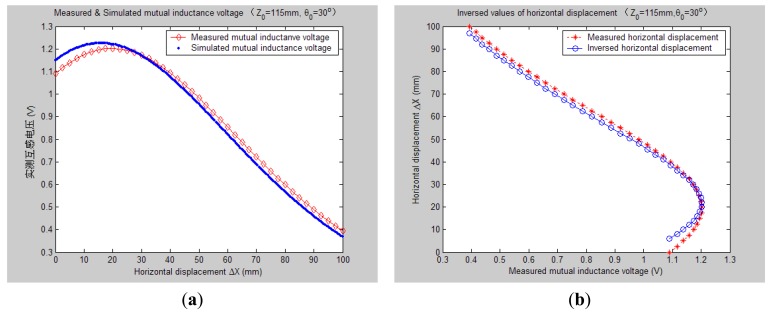
Horizontal displacement inverse results of H-type sensor (*θ*_0_ = 30°, *Z*_0_ = 115 mm).

To analyze the influence of measurement noise for parameter inversion, the comparisons between Figures 7a–13a and Figures 7b–13b have been conducted. It can be seen that the smaller deviation exists between the experimentally measured curves and EELA modeling curves of mutual inductance voltage, or, the less voltage measurement noise occurs, the better curve fitness has achieved between the parameter inversion and experimental measurement of horizontal displacement. For example, when *θ*_0_ is 30°, there exists relatively big measurement deviation between the measured and modeled voltage, the absolute average inversion deviation reaches 2.50 mm. This infers that to achieve the good underground displacement parameter identification, the following basic conditions are required: small measurement noise, reliable parameter inversion algorithm and accurate forward modeling.

As can be seen from Figures 12 and 13, when the tilt angle *θ*_0_ are equal to 25° and 30°, there are relatively obvious deviations between the experimentally measured and EELA simulated mutual inductance voltage. Here we give a brief explanation. The mutual inductance voltage graph shown in Figure 5 is made of seven separate curves according to the different values of *θ*_0_. Before measurement of each mutual inductance voltage curve, it is required that the initial central distance *X*_0_ between two sensing units be adjusted to 0 mm. However, along with the increase of *θ*_0_ it's hard to guarantee adjustment of *X*_0_ to be precise zero, thus causing some voltage measurement error to parameter Δ*X*.

On the other hand, as Figures 12 and 13 illustrate, when *θ*_0_ is large, both the measured and simulated mutual inductance voltage are not decreasing monotonically as the horizontal displacement Δ*X* increases, which further increases the difficulty of the horizontal displacement inversion. For this, we have presented some modified search optimization algorithms into the executive program of the proposed underground displacement inversion methods. Examples include: (1) partition neighborhood search algorithm (*i.e*., to search for the Δ*X* match value concerning the voltage extreme point firstly and then search for Δ*X* predicted values from both sides of the voltage extreme point respectively, according to the search optimization principle); (2) multi-fitting accuracy combination search algorithm (*i.e*., first applies the highest fitting precision criterion to search for match point of Δ*X*, if the search is not successful, then gradually reduces search fitting accuracy under some preset conditions); (3) removing flying spots in measured data to avoid inducing artificial noise after smooth the data. All these have overcome the problem of non-convergence or serious inversion distortion during the inversion process of parameter Δ*X* that might be caused by the non-monotonicity and measuring error of the mutual inductance voltage, thus have improved the parameter inversion accuracy and efficiency.

All these have overcome the problem of non-convergence or serious inversion distortion during the inversion process of parameter Δ*X* that might be caused by the non-monotonicity and measuring error of the mutual inductance voltage, thus have improved the parameter inversion accuracy and efficiency. In order to further evaluate validity and accuracy of the proposed horizontal displacement parameter inversion method for the H-type sensor, we have applied this inversion method to derive a series of the horizontal displacements under different setting values of axial distance *Z*_0_ (such as 110 mm, 115 mm, …, 135 mm) and made comparisons with the counterpart experimentally measured values. These inversion and comparison results show that during the entire process of *Z*_0_ varied from 110 mm to 135 mm (varied by 5 mm intervals), the horizontal displacement inversed values always show quite good fitness with the measured values. More specifically, both the relative and absolute inversion deviation averages are generally controlled within 3 mm when the variation range of measured horizontal displacements is set as 0–100 mm. It further testifies the effectiveness and accuracy of the proposed parameter inversion method to deduce the measuring underground displacement parameter for the H-type sensor.

To provide a direct and close-up comparison to the inversion results when *Z*_0_ is set as 115 mm, Table 3 gives both the relative and absolute inversion deviation averages between the inversed and measured horizontal displacement when *Z*_0_ is set as 125 mm while *θ*_0_ is changed from 0° till 30° with 5° change intervals. Similarly, it can been found that when *θ*_0_ is varied from 0° to 30°, the relative and absolute mean inversion deviations of horizontal displacement are changed between −2.87–2.24 mm and 0.59–3.02 mm, respectively. So it proves again that it is quite reliable and accurate to apply the proposed “EELA forward model-optimization inversion method” to derive the measuring underground horizontal displacement for the H-type sensor.

## 5. Conclusions

Sudden geological disasters cause great harm. They not only seriously threaten the safety of human life, but can also greatly damage the development of human society, economic development and the resource environment. Displacement variation is a direct reflection of the movement and deformation characteristics of geological disaster masses, so underground displacement monitoring is one of the important methods and bases for geological disaster prediction and forecasting.

In our previous studies we designed a simple and novel electromagnetic underground displacement sensor, namely the H-type sensor. It can convert the measuring underground horizontal displacement and tilt angle to the variations of mutual inductance and inclination measuring voltage. Meanwhile, we proposed a quite accurate and efficient mutual inductance voltage measuring model, namely, the EELA model, to describe the functional relationship among the H-type sensor's output of mutual inductance voltage, the measuring parameters of underground horizontal displacement and tilt angle, and its geometry and shape parameters.

Based on the above research, this paper has presented an underground horizontal displacement parameter inversion approach called the EELA forward simulation- optimization inversion method, which has the following features: first, it applies the EELA-based mutual inductance voltage measuring model as the H-type sensor's forward simulation model to generate the simulated signal of mutual inductance voltage. Second, both the simulated signal and measured signal of mutual inductance voltage, together with the sensor measured tilt angle and initial model parameters, are input into the parameter inversion system and applied with the comprehensive optimization inversion algorithm, to realize the inversion of underground displacement parameter for the H-type sensor.

A series of comparative studies between the inversed and the measured values of horizontal displacement when both the tilt angles *θ*_0_ and initial axial distances *Z*_0_ are changed have been conducted. The study results show that the inversion deviation is stable and less than 3 mm when the measured horizontal displacements are varied in 0–100 mm range under different *θ*_0_ and *Z*_0_ conditions, so it is verified that the proposed EELA forward simulation-optimization inversion method is effective for deducing the underground horizontal displacement for the H-type sensor with quite good inversion accuracy and stability.

## Figures and Tables

**Table 1.** Geometrical parameters of Solenoids I and II for the proposed H-type sensor.

**Parameter**	**Unit**	**Value**	**Comment**
Diameter (*d*)	mm	70	
Length (*a*)	mm	75	
Axial distance (*Z*_0_)	mm	115	
Coil turns (*w*)	mm	400	wound by 3 layers

**Table 2.** Comparison of the inversed and the measured values of horizontal displacement (*Z*_0_ = 115 mm).

**Tilt Angle (°)**	**Relative Average Inversion Deviation (mm)**	**Absolute Average Inversion Deviation (mm)**
0	−1.54	1.59
5	−0.59	0.90
10	1.06	1.09
15	−0.24	0.32
20	0.62	0.87
25	0.84	1.72
30	0.60	2.50

**Table 3.** Comparison of the inversed and the measured values of horizontal displacement (*Z*_0_ = 125 mm).

**Tilt Angle (°)**	**Relative Average Inversion Deviation (mm)**	**Absolute Average Inversion Deviation (mm)**	
0	−2.87	2.87	
5	−2.20	2.20	
10	0.335	0.59	
15	−0.652	0.65	
20	0.805	1.29	
25	1.52	2.08	
30	2.24	3.02	
